# Next-generation amplicon sequencing identifies genetically diverse human astroviruses, including recombinant strains, in environmental waters

**DOI:** 10.1038/s41598-018-30217-y

**Published:** 2018-08-07

**Authors:** Akihiko Hata, Masaaki Kitajima, Eiji Haramoto, Suntae Lee, Masaru Ihara, Charles P. Gerba, Hiroaki Tanaka

**Affiliations:** 1 Research Center for Environmental Quality Management, Kyoto University, Shiga, Japan; 2 Department of Urban Engineering, Graduate School of Engineering, The University of Tokyo, Tokyo, Japan; 3 Division of Environmental Engineering, Faculty of Engineering, Hokkaido University, Hokkaido, Japan; 4 Interdisciplinary Center for River Basin Environment, Graduate Faculty of Interdisciplinary Research, University of Yamanashi, Yamanashi, Japan; 5 Department of Soil, Water and Environmental Science, The University of Arizona, Tucson, Arizona USA

## Abstract

Human astroviruses are associated with gastroenteritis and known to contaminate water environments. Three different genetic clades of astroviruses are known to infect humans and each clade consists of diverse strains. This study aimed to determine the occurrence and genetic diversity of astrovirus strains in water samples in different geographical locations, i.e., influent and effluent wastewater samples (n = 24 each) in Arizona, U.S., and groundwater (n = 37) and river water (n = 14) samples collected in the Kathmandu Valley, Nepal, using next-generation amplicon sequencing. Astrovirus strains including rare types (types 6 and 7 classical human astroviruses), emerging type (type 5 VA-astroviruses), and putative recombinants were identified. Feline astrovirus strains were collaterally identified and recombination between human and feline astroviruses was suggested. Classical- and VA-astroviruses seemed to be prevalent during cooler months, while MLB-astroviruses were identified only during warmer months. This study demonstrated the effectiveness of next-generation amplicon sequencing for identification and characterization of genetically diverse astrovirus strains in environmental water.

## Introduction

Astroviruses (AstVs) are classified into the family *Astroviridae* and known to infect a wide range of mammalian and avian hosts including humans^1^. AstVs possess icosahedral capsid with a diameter of 28–31 nm packaging single-stranded positive-sense RNA genome of 6.2–7.8 kb in length^1^. The genome contains three open reading frames (ORFs), i.e., ORF1a, ORF1b, and ORF2. ORF1a and ORF1b encode nonstructural proteins such as serine protease and RNA dependent RNA polymerase, while ORF2 encodes structural proteins^1^. To date, genetically diverse AstV strains have been identified. The strains so called human AstVs are regarded as “classical” AstVs (CAstVs), which are known as an important cause of infantile viral gastroenteritis. Their infections occur mainly sporadically and contribute to 2–10% of gastroenteritis cases^2–4^. Recent investigations identified emerging human AstVs belonging to two clades (MLB- and VA-clades), which are genetically distinct from CAstVs. Genetically diverse strains have also been found within each clade. CAstVs, MLB-AstVs, and VA-AstVs are currently divided into 8, 3, and 5 genotypes, respectively^1,5^. Human AstVs including the novel clades are regarded as enteric pathogens, but recent studies suggested that they also affect the central nervous system^6–8^. Type 1 VA-AstVs are strongly suggested to be associated with encephalitis in immunocompromised patients^7^. Compared to other human enteric viruses such as noroviruses and rotaviruses, human AstVs are not well studied^9^. Therefore, especially for MLB- and VA-AstVs, their characteristics such as prevalence and epidemiology have not been fully elucidated.

Enteric viruses including human AstVs are excreted in the feces from infected individuals and contaminate the water environment directly or via wastewater treatment facilities^10^. Clinical surveillance is widely applied to understand the prevalence of enteric viruses^2,3,5,11–13^. However, enteric virus infections occasionally result in asymptomatic cases that are not usually identified by clinical surveillance^14,15^. Presence of enteric viruses in water environments indicates the presence of infected individuals including those asymptomatically infected and potential infection risk via water. Thus, investigation of viruses in environmental water has significance in view of epidemiology and risk management. During these two decades since the first identification of human AstVs in the water environment in Spain^16^, only a few studies have focused on human AstVs in water^1^. Particularly, presence of MLB- and VA-AstVs in environmental water samples has been reported by only two studies conducted in Japan and Uruguay^17,18^. Both studies revealed that genetically diverse human AstV strains are present in the environment^17,18^. This motivated us to investigate the presence of human AstVs in other regions.

Most of the previous studies analyzed AstV nucleotide sequences in environmental samples using Sanger sequencing, by which limited numbers of sequence reads can be obtained^17–23^. Recently, next-generation sequencing technologies became available, which potentially allows investigation of viral genetic diversity in a given environmental sample with much higher throughput and greater sequencing depth. As previously reported, genetically diverse viral strains are co-present in fecally contaminated water^17,21,24–26^. Next-generation amplicon sequencing can be an ideal technique to determine genetic diversity of a target virus in a water sample in depth.

This study aimed to investigate the prevalence and genetic diversity of human AstVs including the novel clades in water samples in the U.S. and Nepal. We also intended to demonstrate the usefulness of next-generation amplicon sequencing approach to reveal the presence of emerging/rare AstV strains in complex environmental samples.

## Results

### Occurrence of human AstVs in water samples

In this study, we sequenced 43, 7, and 23 of CAstV, MLB-, and VA-AstV positive nested-PCR amplicons, respectively, with three separate NGS runs (Table S1 in the Supplementary Information). A total of 79,957,568 reads were obtained. Among them, 10,367,900, 185,136, and 556,473 reads showed >70% similarity to CAstV, MLB-, and VA-AstV reference strains, respectively. Table 1 summarizes the occurrence of human AstVs belonging to CAstV, MLB-, and VA-clades in waste, ground, and river water samples. From wastewater samples collected in U.S. (n = 24 each for influent and treated effluent samples), CAstVs were identified with relatively high frequencies, 75% (18/24) and 63% (15/24) from influent and treated effluent samples, respectively, while MLB-AstVs were identified in only 4.2% (2/48) of the wastewater samples. VA-AstVs were identified in 54% (13/24) and 21% (5/24) of influent and treated effluent samples, respectively. WWTP-A and -B utilize different treatment processes, i.e., conventional activated sludge process and biological tricking filter process, respectively. There was no clear difference in the detection frequencies of human AstVs in the effluent samples between these two WWTPs; CAstV, MLB-, and VA-clades were identified from 7, 0, and 2 (n = 12 each) effluent samples, respectively, at WWTP-A, and 8, 1, and 3 (n = 12 each) effluent samples, respectively, at WWTP-B.

**Table 1:** Positive rates of each genotype of human astroviruses from each sample matrix.

Clade	Genotype	U.S.		Nepal	
		Influent wastewater	Treated effluent wastewater	Groundwater	River Water
		(*n* = 24)	(*n* = 24)	(*n* = 37)	(*n* = 14)
CAstV^a^	Type 1	16	11	1	7
	Type 2	11	6	1	5
	Type 3	4	4	0	3
	Type 4/8	18	13	1	6
	Type 5	10	6	1	5
	Type 6	2	1	0	4
	Type 7	2	1	0	6
	FAstV^b^	5	6	1	0
	Putative recombinant^c^	4	1	0	0
	Any type (% positive)	18 (75%)	15 (63%)	2 (5.4%)	8 (57%)
MLB-AstV	Type 1	1	1	0	4
	Type 2	1	0	0	3
	Type 3	0	0	1	3
	Putative recombinant	0	0	0	2
	Any type (% positive)	1 (4.2%)	1 (4.2%)	1 (2.7%)	4 (29%)
VA-AstV	Type 1	10	2	0	4
	Type 2	10	3	0	4
	Type 3	9	2	0	1
	Type 4	0	0	0	0
	Type 5	0	0	0	1
	Any type (% positive)	13 (54%)	5 (21%)	0 (0.0%)	5 (36%)

^a^CAstV, classical human astrovirus.

^b^FAstV, feline astrovirus.

^c^Number of OTU that were suggested to be recombinant by Simplot analysis.

Groundwater samples collected in Nepal (n = 37) resulted in low detection frequencies for human AstVs, only 2 (5.4%) and 1 (2.7%) samples were positive for CAstV and MLB-AstV, respectively. River water in Nepal (n = 14) showed 57% (8/14), 29% (4/14), and 36% (5/14) detection frequencies of CAstV, MLB-AstV, and VA-AstV, respectively.

### Genotype distribution of human AstVs

Genotypes of human AstVs identified in the samples were determined based on phylogenetic analysis (Table 1 and Fig. S1 in the Supplementary Information). As previously reported by other researchers^27^, types 4 and 8 CAstVs are not distinguishable based on our analysis because their nucleotide sequences are closely related at our target regions, 5′-end of ORF2^28^. Due to long amplicon sizes after (semi-)nested PCR, sequencing analysis for MLB- and VA-AstVs was conducted based on partial (5′- and 3′ ends) sequences of the amplicons. Types 1, 2, 4/8, and 5 CAstVs were identified from all sample groups. Types 3, 6, and 7 were also identified from samples other than groundwater samples with lower frequency. Interestingly, in addition to the CAstV strains, feline AstV (FAstV)-like sequences, which formed a distinct cluster from human AstVs and showed the highest homologies of 89–96% with FAstV based on the BLAST analysis (https://blast.ncbi.nlm.nih.gov), were also identified from samples from the U.S. and Nepal. We identified 11 operational taxonomic units (OTUs) that are not classified into clusters with referential sequences.

Only types 1 and 2 MLB-AstVs were identified from the wastewater samples collected in the U.S., whereas all known genotypes of MLB-AstVs (types 1–3) were found from the samples collected in Nepal. Similar to CAstVs, 5 OTUs found in river water samples in Nepal were not classified into clusters with referential sequences.

Types 1–3 VA-AstVs were identified from the wastewater samples collected in the U.S. In addition to types 1–3, type 5 VA-AstVs were identified from river water samples collected in Nepal.

### Identification of putative recombinants

**Figure 1:**
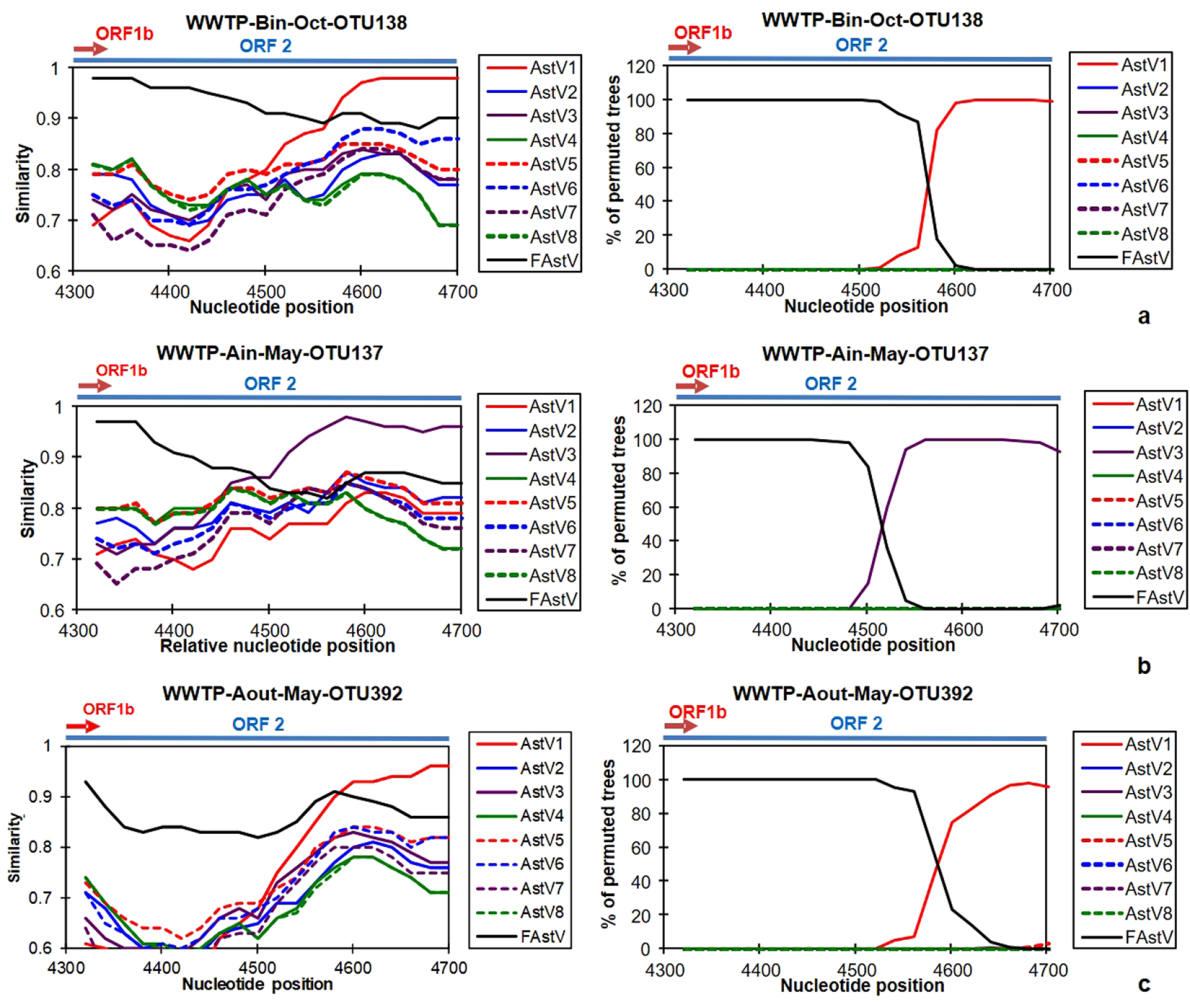
Simplot (left) and bootscan (right) analysis of CAstV OTUs, including FAstV-like OTUs, identified from U.S. samples. OTUs suggested to be recombinants by the phylogenetic analysis (Fig. S1) were subjected to the analysis here. Representative OTUs resulted in 100% bootstrap values among 100 replicates with two different reference strains at the 5′ and 3′ end in the bootscan analysis are shown here. Results obtained from other OTUs are shown in Fig. S2 in the Supplementary Information. Names of OTUs used as a query sequence in each analysis are shown on upper side of the figures by the following manner, “Origin of the sample (- “type of wastewater”)” - “year or month of sample collection”- “OTU number”. A” and “B” indicate places of WWTP and “in” and “out” indicate influent and treated effluent, respectively. GenBank accession numbers of referential strains are as follows, CAstV type 1: L23513; -2: L13745; -3: AF141381; -4: DQ344027; -5: DQ028633; -6: GQ495608; -7: AF248738; -8: AF260508; and FAstV: KM017742. Window size and step size for each analysis were set as 100 and 20 nt, respectively. Nucleotide position shown on x-axis corresponds to CAstV type 1 strain Oxford (GenBank accession number: L23513). Arrows and lines indicate locations of ORF1b and ORF2.

**Figure 2:**
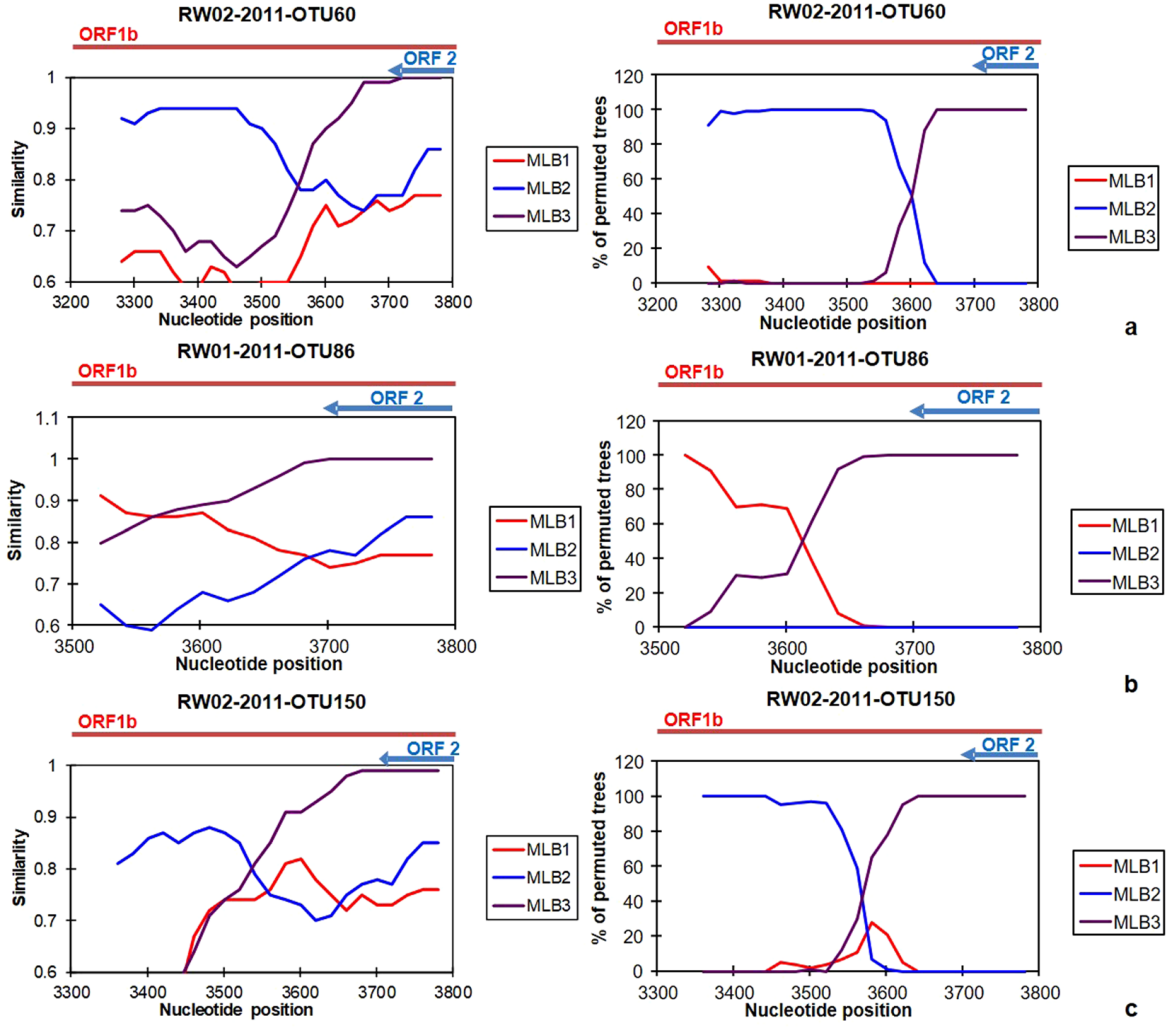
Simplot (left) and bootscan (right) analysis of MLB-AstV OTUs identified from Nepal samples. OTUs suggested to be recombinants by the phylogenetic analysis (Fig. S1) were subjected to the analysis here. Representative OTUs resulted in 100% bootstrap values among 100 replicates with two different reference strains at the 5′ and 3′ end in the bootscan analysis are shown here. Results obtained from other OTUs are shown in Fig. S2 in the Supplementary Information. Names of OTUs used as a query sequence in each analysis are shown on upper side of the figures by the following manner, “Origin of the sample (- “type of wastewater”)” - “year or month of sample collection”- “OTU number”. GenBank accession numbers of referential strains are as follows, MLB-AstV type 1: FJ222451;-2: JF742759; and-3: JX857870. Window size and step size for each analysis were set as 100 and 20 nt, respectively. Nucleotide position shown on x-axis corresponds to MLB-AstV type1 (GenBank accession number: FJ222451). Arrows and lines indicate locations of ORF1b and ORF2.

Eleven and five OTUs obtained from CAstV in U.S. and MLB-AstV in Nepal samples, respectively, were not classified into clusters with referential sequences (Fig. S1). We assumed that the deviated OTUs are recombinants and carried out Simplot analysis to investigate whether recombination is identified on their genomes. As a result, all the unclassified OTUs were suggested to be recombinant based on the Simplot analysis. Among the putative recombinants, those supported by high bootstrap values (100% among 100 replicates) by the bootscan analysis with two different reference strains at the 5′ and 3′ ends are shown in Fig. 1 and -2 and others supported by lower bootstrap values (75–100%) are shown in Fig. S2. Putative recombinations of CAstV were observed not only among CAstV strains (type 4/8 and type1 (Fig. S2A7) and type 4/8 and type 3 (Fig. S2. A8)), but also between CAstV and FAstV strains (type 4/8 or type 2 and FAstV, Fig. 1, Fig. S2A1–6). Regarding MLB-AstV OTUs, all potential recombination events occurred between type 3 MLB-AstV and type 2 or -1 (Figs 2 and S2B).

### Seasonal distribution of human AstVs

Table 2 summarizes numbers of OTUs of each AstV clade obtained from wastewater samples (U.S.) in each sampling month. Wastewater samples in U.S. were collected monthly for a year. The frequency of CAstV identification during cooler months, November to April, was significantly higher (96%, 23/24) than that during the rest of the months (42%, 10/24) (chi-square test, *p* < 0.01) (Fig. S1 and Table 2). FAstV-like OTUs were also more frequently identified during cooler months. Similarly, the frequency of VA-AstV identification during the cooler months was higher (50%, 12/24) than that during rest of the months (25%, 6/24), even though the difference was not statistically significant (chi-square test, *p* = 0.14) (Fig. S1 and Table 2). MLB-AstV was identified only in May and June (Fig. S1 and Table 2).

**Table 2:** Number of OTUs of each human AstV clade determined from wastewater samples in U.S.

	WWTP	2011					2012						
		Aug	Sept	Oct	Nov	Dec	Jan	Feb	Mar	Apr	May	June	July
CAstV	A-in^b^			14	56	14	58	32	116	27	5	4	
	A-out				72	72		44	18	55	167	4	
	B-in	1	1		57	36	29	17	33	18		7	
	B-out		36		16	20	6	8	30	6	10		
MLB-AstV^a^	A-in											11–29	
	A-out												
	B-in												
	B-out										24–11		
VA-AstV	A-in				1-1			9-2	24-16	21-20	30-21	11-7	43-17
	A-out							16-14		13-21			
	B-in				3-1	8-3	7-3	12-13		36-27	12-13		
	B-out			1-2	1-0						8-9		

^a^Number of MLB- and VA-AstVs OTUs were shown as follows, “number of OTUs formed from 5′- end of amplicons sequences – those formed from 3′-end of amplicons sequences”.

^b^“A” and “B” indicate places of WWTP. “in” and “out” indicate influent and treated effluent, respectively.

## Discussion

In this study, we successfully identified human AstVs, including emerging MLB- and VA-AstVs, in water samples collected from two geographically distinct countries, U.S. and Nepal. On the contrary to its importance as an enteric pathogen, presence of AstVs in water is not as frequently documented as other enteric viruses like noroviruses and rotaviruses^9^. MLB- and VA-AstVs are emerging viruses that were discovered in 2008 and 2009, respectively, and only limited information is available about them^13,29,30^. Wastewater and environmental water contain viruses excreted from infected individuals including those asymptomatically affected^15^; thus, investigation of viruses in water can reveal genetic diversity of circulating virus strains in the study area.

Our groundwater samples in Nepal resulted in lower positive ratios of human AstVs than other samples. Previous studies reported that the groundwater samples were less contaminated with other enteric viruses, i.e., human adenoviruses, noroviruses of genogroup I and II, and Aichi virus 1, than the river water samples^31–33^. All groundwater samples positive for human AstVs were collected from one dug well, which was highly contaminated with waterborne pathogens^32,33^.

Except for groundwater samples, which showed extremely low human AstV detection frequencies, CAstVs tended to show the highest detection frequencies regardless of the sample types. VA-AstV showed apparently higher detection frequency than MLB-AstV in wastewater samples in U.S., while these two clades of human AstVs were detected in comparable frequencies from river water in Nepal. In our previous study investigating the occurrence of AstVs in wastewater in Japan, MLB-AstV showed higher detection frequency than VA-AstV^17^. Some clinical studies compared the abundance of MLB- and VA-AstVs in feces from diarrheal patients, although the number of positive specimens is limited^34^. In U.S., MLB-AstVs (3/466 positives) were more prevalent than VA-AstVs (1/466 positive) in St. Louis, while only VA-AstVs (1/196 positive) were identified in Seattle^35,36^. In South Asian countries, only VA-AstVs were identified in Nepal and Pakistan (3/190 and 2/43 positives, respectively), while MLB-AstVs were more prevalent (9/416 positive) than VA-AstVs (3/416 positives) in India^13,35^. These imply that relative abundance of MLB- and VA-AstVs in water and clinical specimens varies depending on geographical location. It is noteworthy that in all the previous studies in U.S. and South Asia, CAstVs were more predominantly identified than MLB- and VA-AstVs^13,35,36^.

Diversity of human AstV strains in water samples was studied by Sanger sequencing in previous studies^17–19,21–23^, except for one study applying next-generation sequencing^37^. Especially, diversities of MLB- and VA-AstVs in water samples were studied by only one^17^ and two studies^17,18^, respectively. Therefore, only a limited number of genotypes/strains have been identified from water samples. In the present study, we applied next-generation amplicon sequencing and successfully identified diverse CAstV, MLB-, and VA-AstV strains.

CAstV is divided into 8 genotypes based on nucleotide sequence of ORF2^1^. In general, types 6 and 7 CAstVs are rarely identified in clinical and water samples^17,23,37,38^. In this study, we identified these rare genotypes of CAstV even though their detection frequencies were lower than other genotypes. This is probably because of the application of the next-generation sequencing technique, which can read millions of sequences in parallel and therefore enables the identification of minor strains in a sample. These rare genotypes were found from samples collected from both U.S. and Nepal, indicating that rare genotypes of CAstVs are also circulating worldwide. A previous study applied the next-generation amplicon sequencing technique to identify CAstV genotypes in wastewater samples in France^37^. This previous study identified lower number of genotypes (types 1, 2, 5, and 6) than the present study. One reason for the difference may be PCR assays. The previous study applied single-round PCR with primers MON270 and 269^37^, which were designed in 1995^11^. In this study, we applied nested PCR with primers designed in 2014 based on updated list of AstV strains including contemporary circulating ones^27^. Thus, our assay should be more suitable in identifying broad range of CAstVs sensitively.

Not only CAstV but also FAstV-like sequences were identified in our investigation. FAstVs (mammalian AstV-2) are more closely related to CAstVs (mammalian AstV-1) than any other AstV species^1^. Accordingly, our reverse transcription (RT)-nested PCR primers showed only 3-base mismatches with a FAstV sequence in the database (GenBank, KF499111), and therefore, it is highly possible that the assay amplifies FAstV genomes as well. A previous study employing another set of primers for detection of CAstV also found FAstV-like sequence from surface water in urbanized area of Singapore^23^. Unexpected amplification of FAstV gene by primers targeting CAstV may be a common issue and may lead to overestimation of the presence of CAstVs, especially in studies investigating the effect of human fecal contamination of environmental samples. In accordance with previous studies targeting both clinical and environmental samples^1,17,37,39^, CAstV became prevalent during cooler months in this study. FAstV-like sequences also followed the same seasonal pattern, although the number of positive sample is too small to conclude. This suggests that FAstV is closely related to CAstV in terms of genetic distance and seasonal pattern. In a previous study, FAstV has been detected from a domestic cat^40^. Besides, another previous study has revealed that canine kobuvirus is present in municipal wastewater^41^. These suggest that viruses excreted from pet animals can contaminate water environment with human viruses. It is possible that cats in the study area are the source of FAstV-like sequences identified in this study.

Two previous studies have investigated the presence of MLB-AstVs in water samples^17,18^. Each study identified one genotype, i.e., one study identified type 2^17^ and the other identified type 1^18^. In the present study, multiple types of MLB-AstVs were identified. All three types of MLB-AstVs were found from samples in Nepal, while type 3 was not found from wastewater in U.S. Our findings suggest that the genotype distribution of MLB-AstVs depends on geographical regions. In our wastewater samples, MLB-AstVs were identified only in early summer (May and June). On the contrary, our previous study suggested that MLB-AstV become prevalent during winter in wastewater in Japan^17^. This implies that seasonal pattern of MLB-AstVs varies depending on geographical regions, although it is not conclusive. It is important to note that the previous study resulted in extremely high detection frequency probably because the study investigated samples collected during only cooler months^17^.

To the best of our knowledge, this is the first study identifying types 3 and 5 VA-AstVs in water samples. Type 5 VA-AstV is a newly identified type, discovered from a pediatric stool sample from Gambia^5^. Our result demonstrates that type 5 VA-AstVs are also circulating in Nepal. In a previous study, type 4 VA-AstV was identified from stool samples in Nepal^41^; however, this genotype was not identified in the present study. Analysis of wastewater samples suggested that seasonal pattern of VA-AstV is similar to that of CAstV, become prevalent during colder month, but the epidemic period seemed to be shorter than that of CAstV. Such a seasonal pattern of VA-AstV is not conclusive. Future establishment of quantitative assay for VA-AstV and long-term monitoring should provide more insights.

It has been suggested that recombination events play an important role in the evolution of AstVs^43^. We identified some potential recombinant strains in the present study. Our RT-nested PCR assay targeting the ORF1b-ORF2 junction region, which is known as a “hotspot” of recombination, allowed us to identify potential recombinant strains^43,44^. Some CAstV genomes were considered potential recombinants. The most frequently reported recombination breakpoints are nucleotide positions between 4100 and 4400 in CAstV-1 (GenBank accession number: L23513), just around the ORF1b-ORF2 junction region^43–46^. Most of CAstV recombination breakpoints suggested by our analysis are nucleotide positions between 4500 and 4600, which are downstream of those frequently reported. To our knowledge, recombination events at the area were not reported in previous studies but those at positions apart from the junction region are possible^43,47^.

Interestingly, recombination between human and FAstV was also identified. The recombination between human and feline strains suggests zoonotic transmission of AstVs, which may lead to an emerging and/or unrecognized risk. Indeed, CAstVs have been detected from non-human primates and piglet samples^48,49^, and evidences of recombination between human- and these animal AstVs were concurrently reported^48,49^. Future investigation of zoonotic potential of AstVs is required. Recombination within MLB-AstV strains, which has not been reported in previous studies, was also suggested in this study. Our findings can prompt future investigations of recombination events occurring among broad range of AstVs.

Water samples impacted by wastewater potentially contain a variety of viruses originated from a wide number of people. Next-generation sequencing is an ideal technology to identify diverse viruses in a water sample because of its ability for massive parallel sequencing. In the present study, we successfully determined diverse AstV sequences including rare types (types 6 and 7 CAstVs), an emerging type (type 5 VA-AstV), and recombinants by applying next-generation amplicon sequencing approach. The recombinant of CAstV and FAstV strongly suggests zoonotic potential of this AstV strain. The present study also demonstrated that genetically diverse AstVs are circulating in the studied geographical regions.

## Methods

### Sample collection and concentration

Influent (n = 24) and effluent (n = 24) wastewater samples were collected from two wastewater treatment plants (WWTPs, WWTP-A and -B) in Arizona, U.S. monthly between August 2011 and July 2012, as described in Kitajima *et al*.^50^. One of the WWTPs utilized a conventional activated sludge process and the other utilized a biological tricking filter process. An adsorption-elution method followed by ultrafiltration^51^ was used to concentrate 100 and 1,000 mL of influent and treated effluent, respectively, to obtain a final volume of approximately 650 μL.

Groundwater (GW, n = 37) and river water (RW, n = 14) samples were collected from 15 and 8 sites, respectively, between August 2009 and May 2011 in the Kathmandu Valley, Nepal as described previously^31–33^. An electronegative membrane vortex method^52^ was applied to ground and river water samples to obtain 12 mL of virus concentrates. The volumes filtered were 50 or 100 mL for river water and 1,000 mL for groundwater samples except for one sample that allowed filtration of only 50 mL.

### RNA extraction and RT-(semi-)nested-PCR

Viral RNA in the virus concentrates obtained in U.S. and Nepal was extracted using a ZR Viral DNA/RNA Kit (Zymo Research, Irvine, CA, USA) and QIAamp Viral RNA Mini Kit (Qiagen, Hilden, Germany), respectively. Extracted RNA was subjected to RT using a High Capacity cDNA Reverse Transcription Kit (Thermo Fisher Scientific, Waltham, MA, USA), according to the manufacturer’s protocol. Subsequently, (semi-)nested PCR assays targeting the ORF1b-ORF2 junction regions of CAstV, MLB-AstV, and VA-AstV were performed separately as described previously^17^ (Table S2 in the Supplementary Information). Amplification of each target gene was confirmed by visualization under a UV lamp after electrophoresis in a 1.5% agarose gel stained by GelRed^TM^ (Wako, Osaka, Japan). In the first-round PCR, primers AHAstVF1 and AHAstVR1, SF0073 and AHMLBR1, and AHVAF1 and AHVAR1 were used to amplify CAstV, MLB-, and VA-AstV genes, respectively. In the second-round PCR, primers AHAstVF2 and AHAstVR2, F0073 and AHMLBR2, and AHVAF2 and AHVAR2 were used to amplify the first PCR amplicons of CAstV, MLB-, and VA-AstV genes, respectively. Resultant second-round PCR amplicon sizes of CAstV, MLB-AstV, and VA-AstV genes were expected to be 407, 689, and 663 bp, respectively.

### Next-generation amplicon sequencing

The second-round PCR amplicons with expected size for each clade of human AstVs were sequenced with the Illumina MiSeq platform (Illumina, San Diego, CA, USA). Briefly, a sequencing reaction mixture was prepared using the TruSeq DNA LT sample preparation kit (Illumina), and loaded on a MiSeq Reagent Kit v3 (600 cycles) (Illumina), according to the manufacturer’s instructions, which allows the system to obtain read length of up to 300 bp. Nucleotide sequencing was performed for 301 cycles for both ends. Obtained sequencing data were trimmed using CLC Genomics Workbench 7.0 software (CLC bio, Aarhus, Denmark). The trimmed sequence reads obtained from CAstV were paired. Those obtained from MLB- and VA-AstVs were not paired because their expected amplicon sizes (689 and 663 bp, respectively) were longer than the length that can be read by the system. Then, edited sequence reads that showed 70% or higher nucleotide identities to the reference CAstV, MLB-AstV, or VA-AstV strains (Oxford strain: GenBank acc. no. L23513; MLB1 strain: FJ222451; and VA1 strainFJ973620, respectively) were extracted using “Map Reads to Reference” command of the CLC Genomics Workbench 7.0 software and the extracted sequences were subjected to further analysis. The trimmed sequence reads sharing 97% or higher nucleotide sequence identities were assigned in an OTU. Representative sequences from each OTU were used for phylogenetic analysis and genotyping. As mentioned above, sequence reads obtained from MLB- and VA-AstVs were not paired due to their long amplicon sizes. Thus, phylogenetic analysis for MLB- and VA-AstVs were conducted based on partial (5′- and 3′ ends) sequences of the amplicons. OTUs that were not classified within any known phylogenetic clades were analyzed for recombination with the Simplot software version 3.5.1^53^ (http://sray.med.som.jhmi.edu/SCRoftware/simplot/). For phylogenetic analysis, OTUs were aligned using Clustal W program version 1.83 (http://clustalw.ddbj.nig.ac.jp/top-e.html). The distances were calculated by Kimura’s two-parameter method^54^ and phylogenetic dendrograms from bootstrap analysis with 1,000 replicates were generated by the neighbor joining-method.

### Statistical analysis

Chi-square test was performed to determine seasonality of human AstVs in wastewater samples in U.S. using R software version 3.4.0 (https://www.r-project.org/). *P*-values of < 0.01 were considered statistically significant.

## Electronic supplementary material

Supplementary Information
