# Supplementary material for: Next-generation amplicon sequencing identifies genetically diverse human astroviruses, including recombinant strains, in environmental waters
Source: Sci Rep. 2018 Aug 7;8:11837. doi: 10.1038/s41598-018-30217-y (PMC6081416; doi:10.1038/s41598-018-30217-y)
Supplement: Supplementary file 1 — Supplementary Information [file 41598_2018_30217_MOESM1_ESM.docx]

**Supplementary Information**

**Next-generation amplicon sequencing identifies genetically diverse human astroviruses, including recombinant strains, in environmental waters**

Akihiko Hata^1, 2*^, Masaaki Kitajima^3^, Eiji Haramoto^3^, Suntae Lee^1^, Masaru, Ihara^1^, Charles P. Gerba^5^, and Hiroaki Tanaka^1^

*^1^Research Center for Environmental Quality Management, Kyoto University, Shiga, Japan*

*^2^Department of Urban Engineering, Graduate School of Engineering, The University of Tokyo, Tokyo, Japan.*

*^3^Division of Environmental Engineering, Faculty of Engineering, Hokkaido University, Hokkaido, Japan*

*^4^Interdisciplinary Center for River Basin Environment, Graduate Faculty of Interdisciplinary Research, University of Yamanashi, Yamanashi, Japan*

*5Department of Soil, Water and Environmental Science, The University of Arizona, Arizona, U.S.A.*

***Corresponding author:** Akihiko Hata

**Mailing address:** *Rm809, Eng. 14^th^ Bldg, 7-3-1 Hongo, Bunkyo-ku, Tokyo, 113-8656, Japan*

**Tel:** +81-3-5841-6252 **Fax:** +81-3-5841-6252

**E-mail:** [hata@env.t.u-tokyo.ac.jp](mailto:hata@env.t.u-tokyo.ac.jp)

**Running title:** Genetic diversity of human astroviruses in the environments

**SUPPLEMENTAL FIGURES**

| **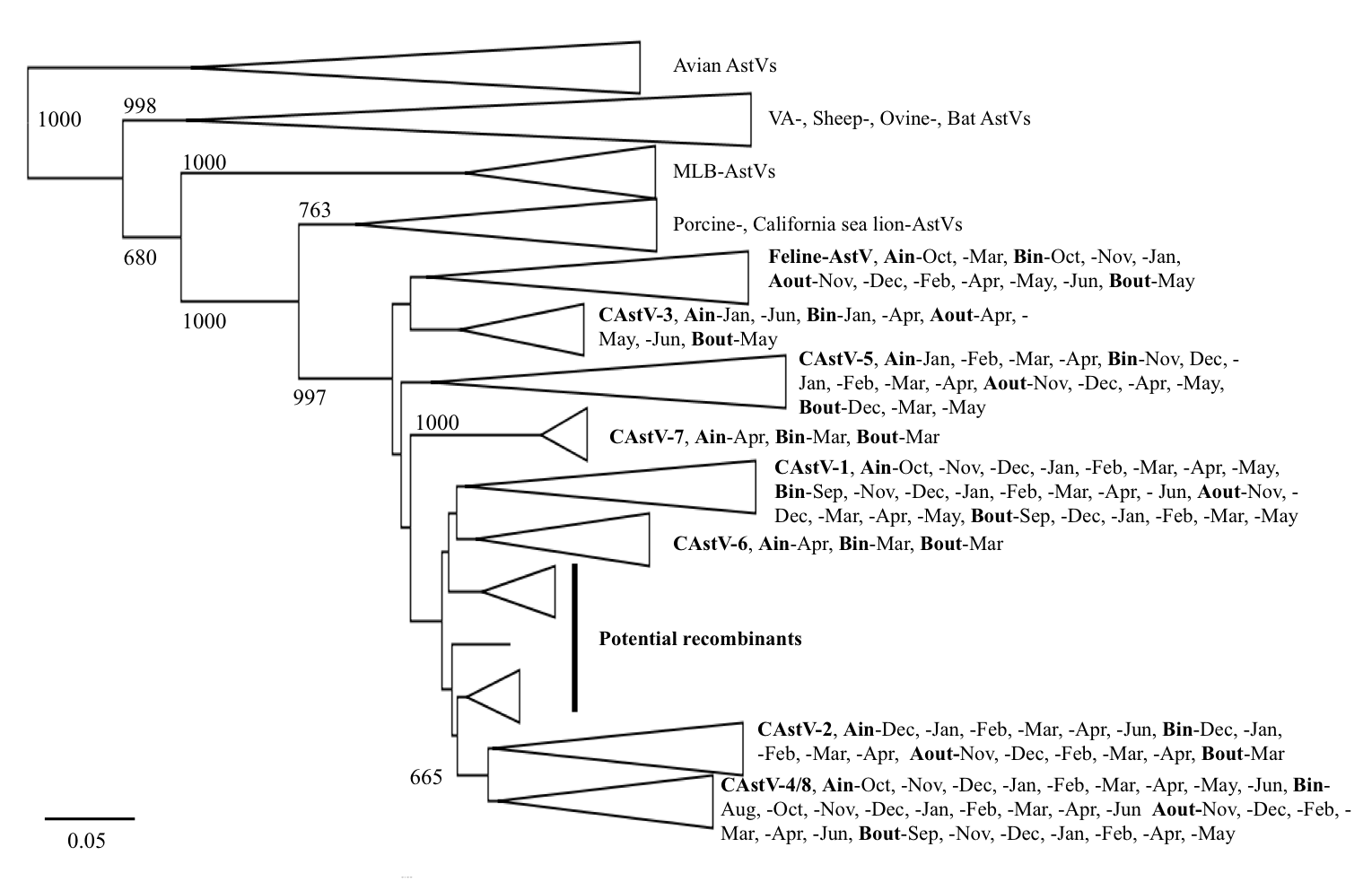** |
| --- |
| **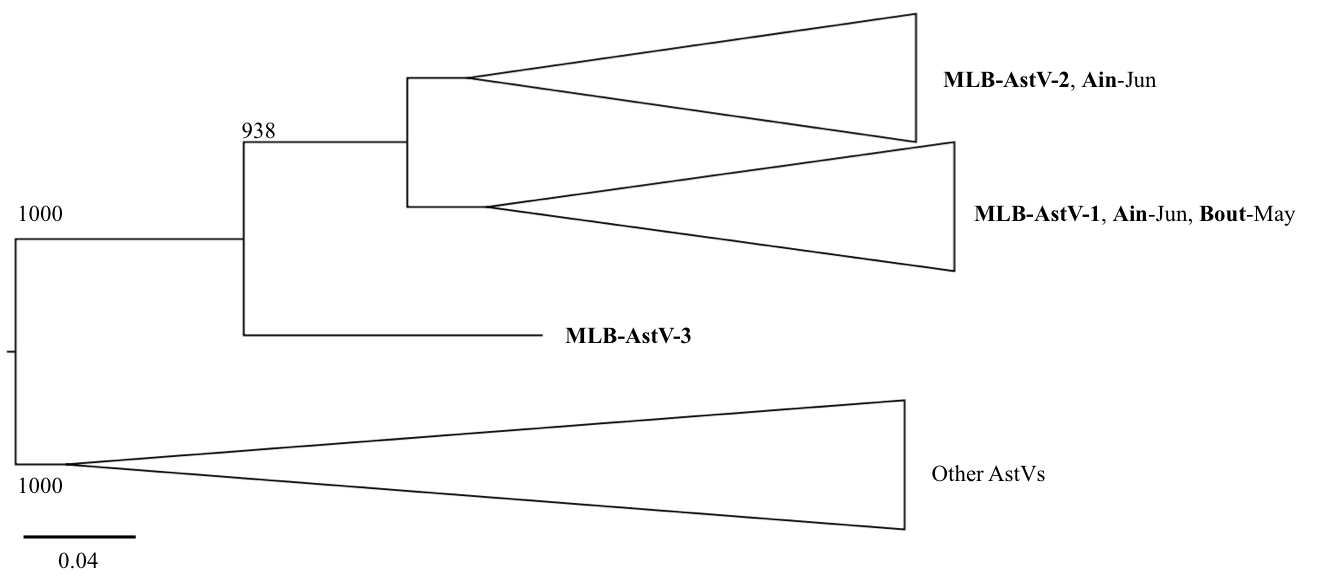**  **A-1**  **A-2** |
| **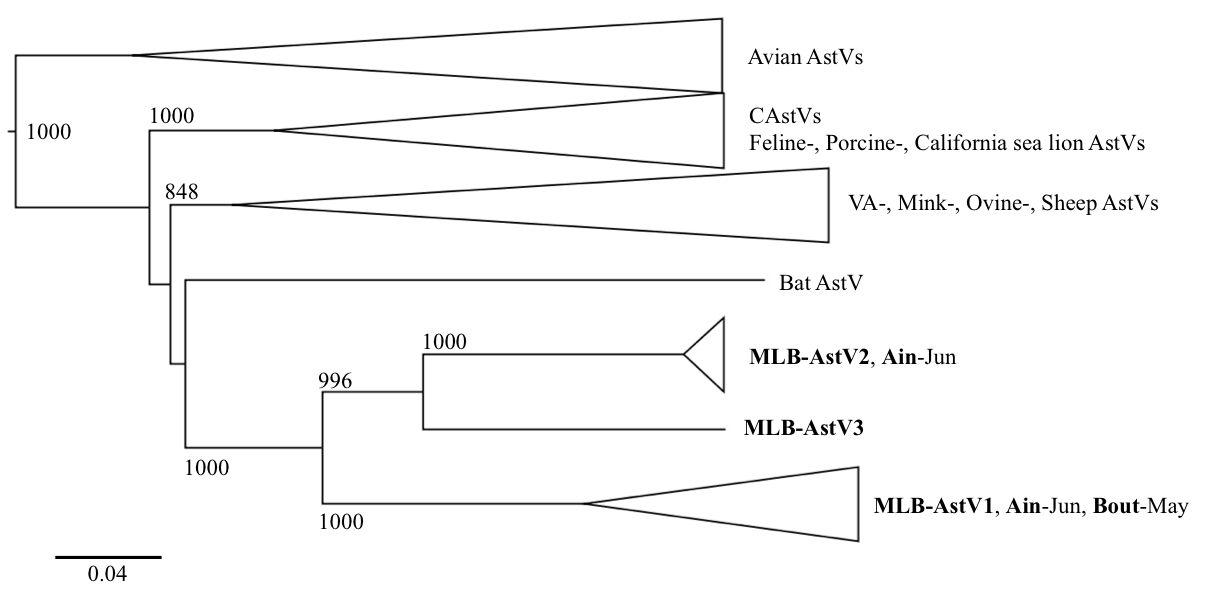**  **A-3** |
| **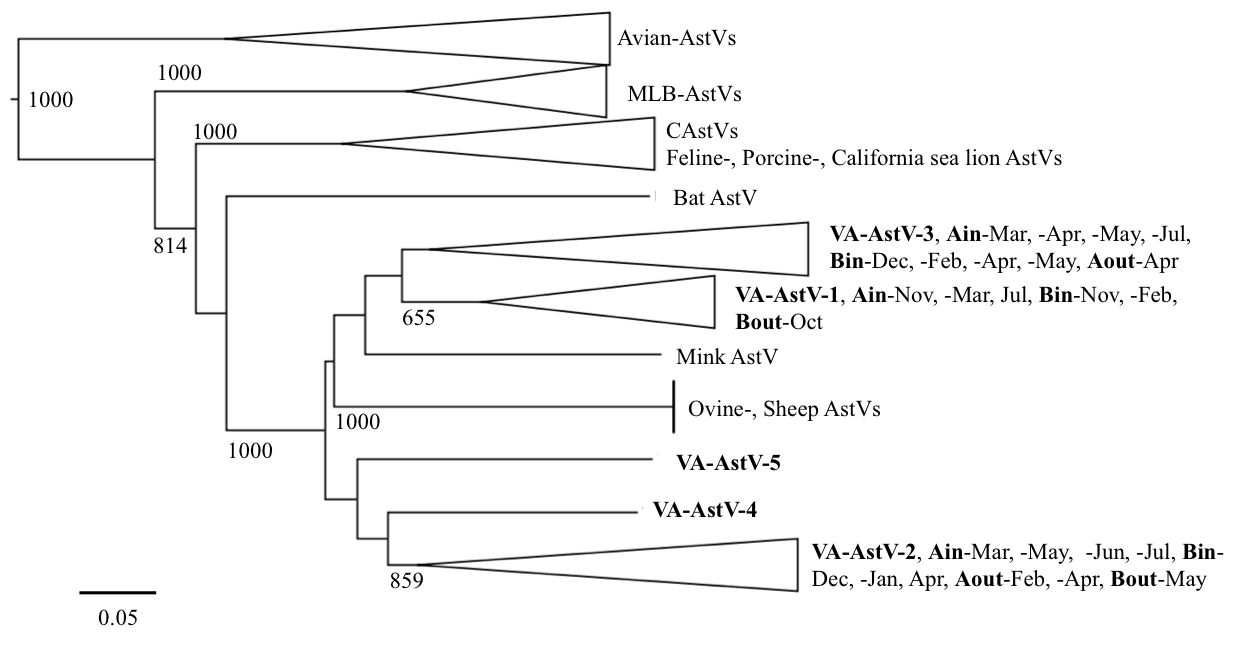**  **A-4** |
| **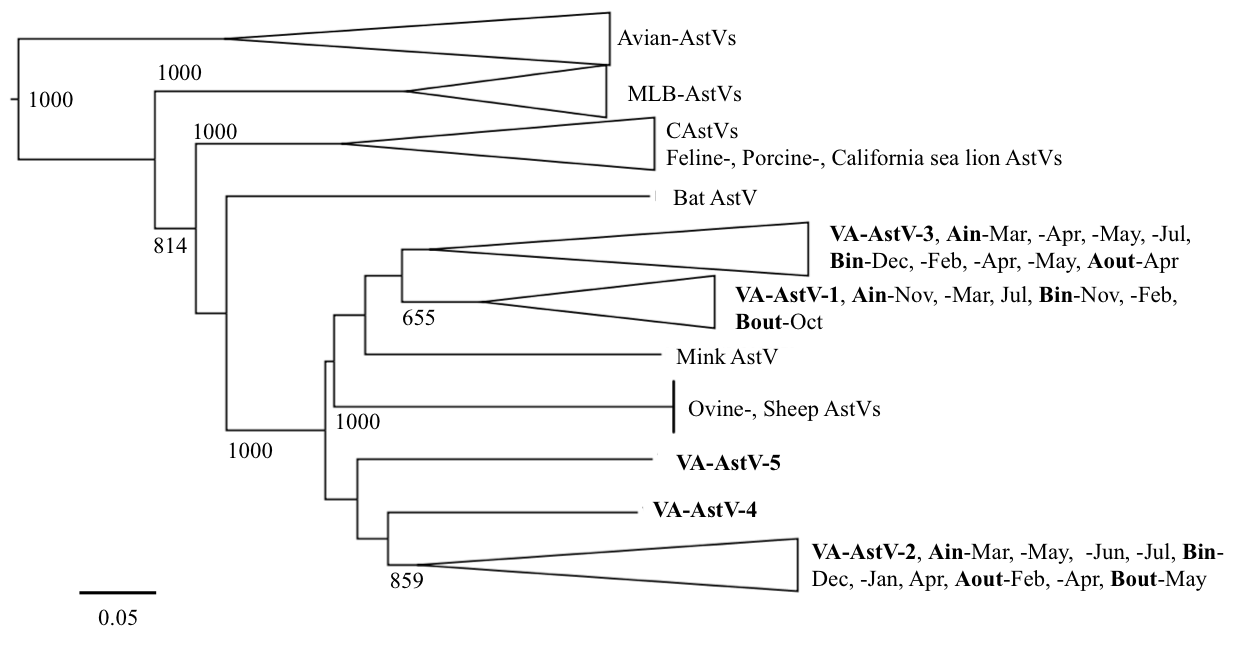** |
| **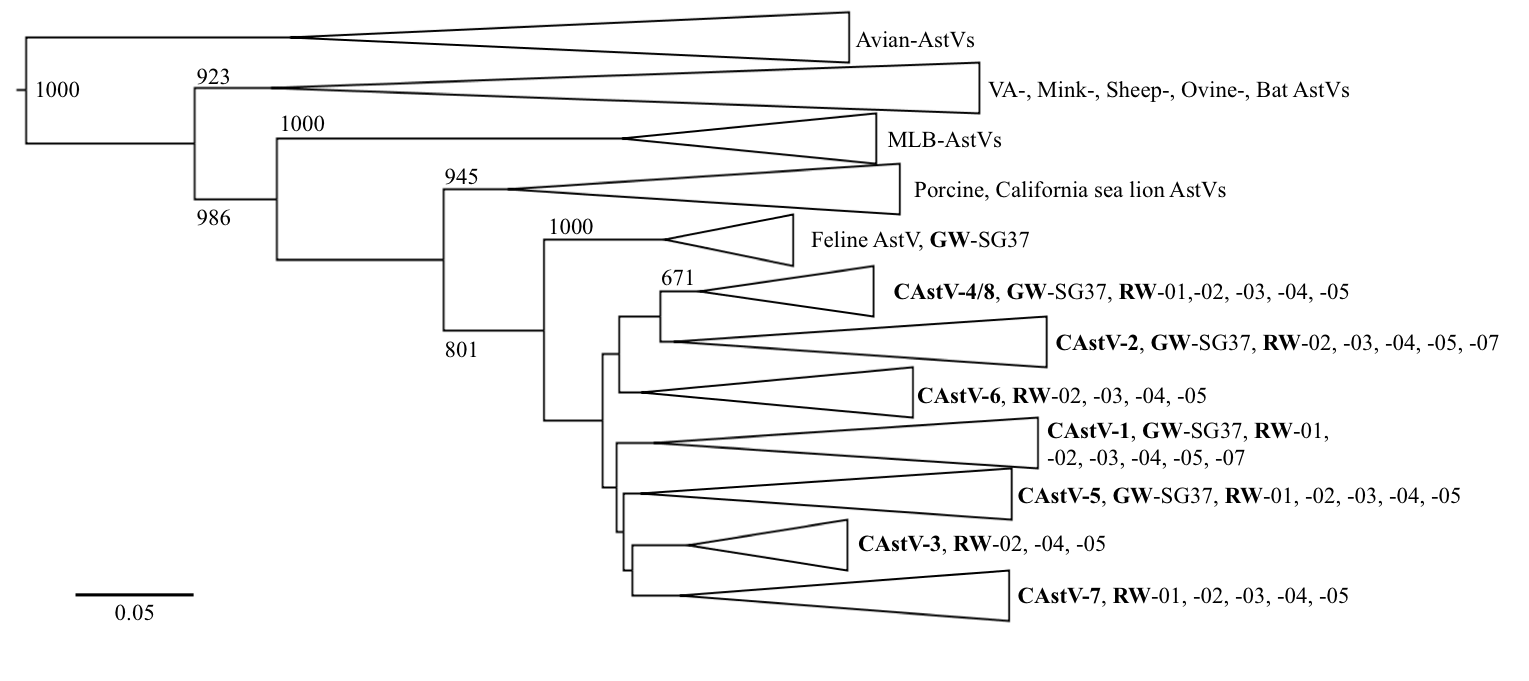**  **B-1**  **A-5** |
| **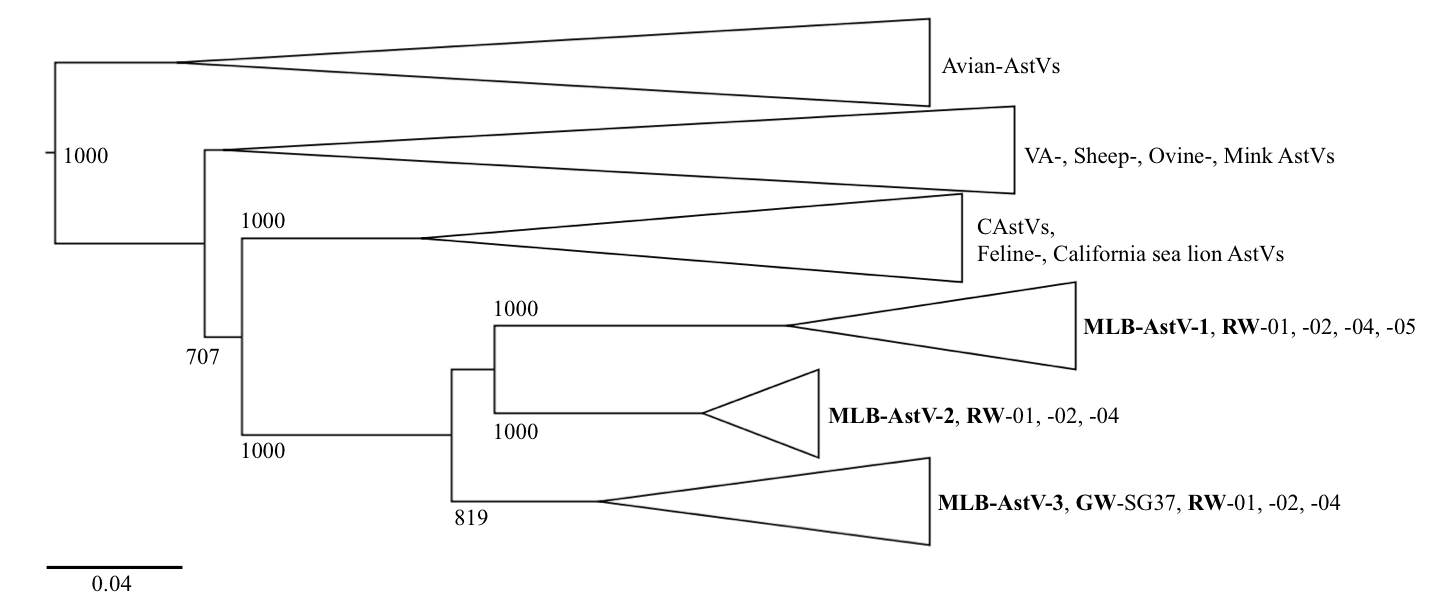**  **B-2** |
| **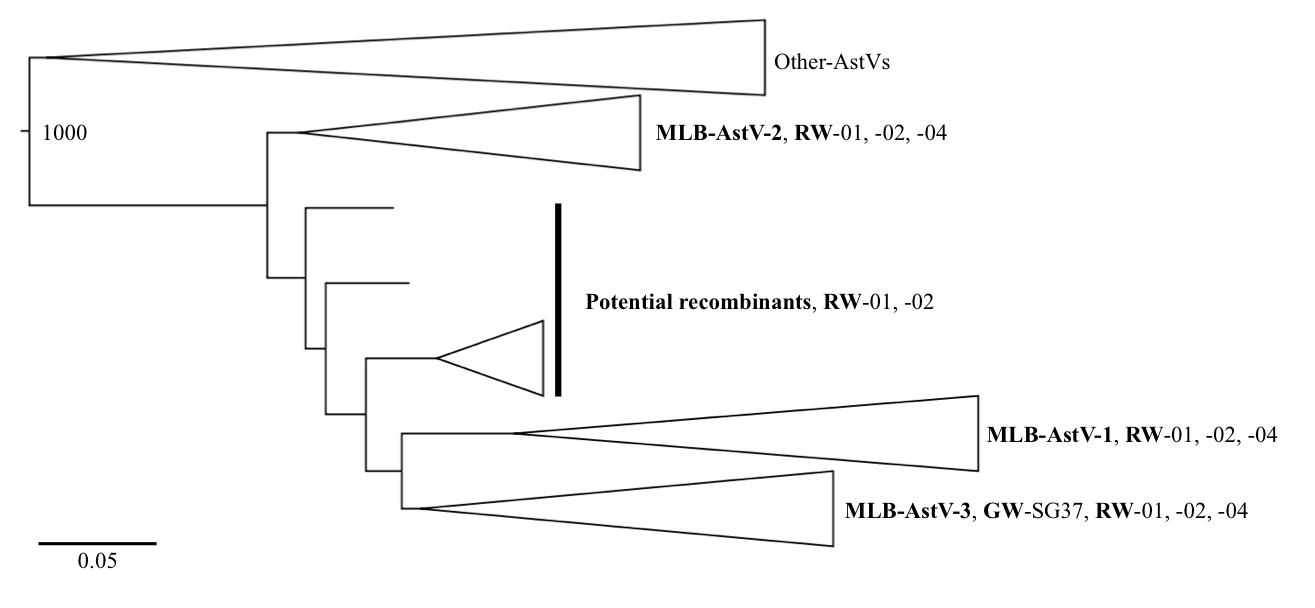**  **B-3** |
| **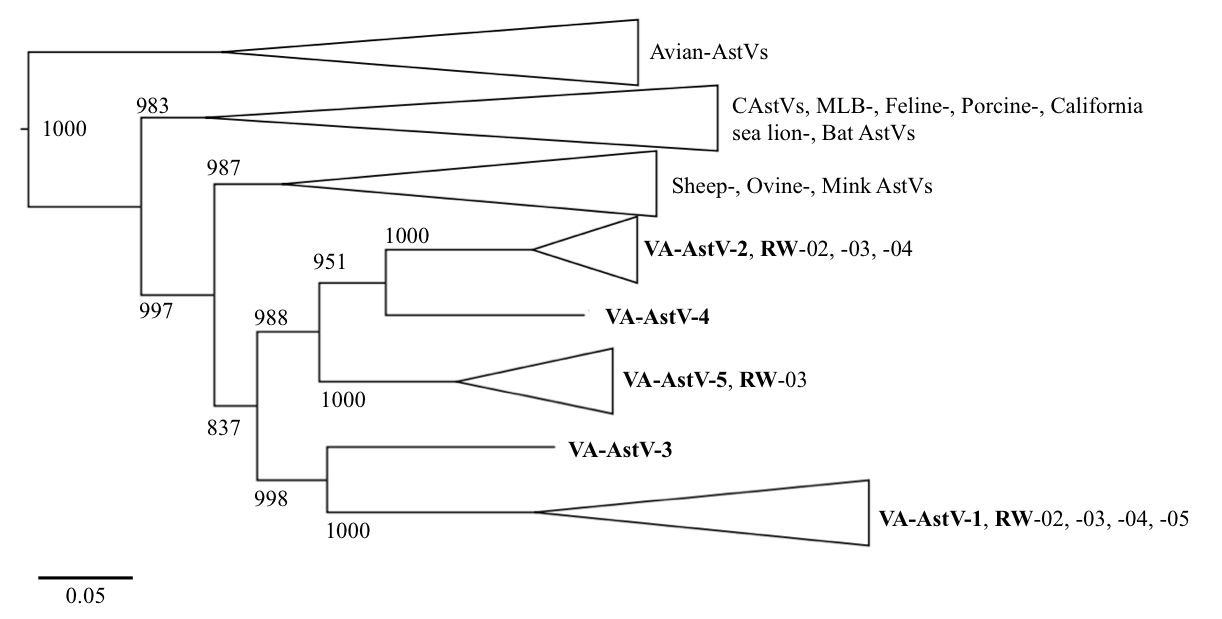**  **B-4** |
| **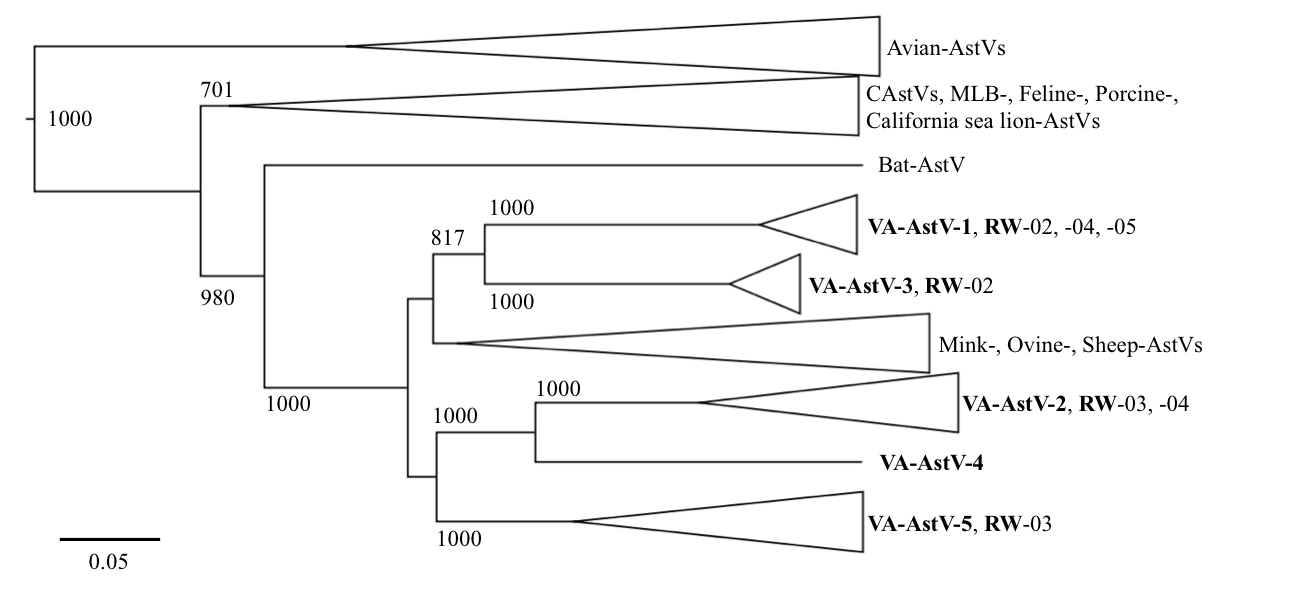**  **B-5** |

**Fig. S1.** Phylogenetic trees of CAstV (A-1 and B-1, approximately 360 nt), MLB-AstV (A-2, A-3, B-2, and B-3), and VA-AstV (A-4, A-5, B-4, and B-5) OTUs identified from U.S. sample group (A) and Nepal sample group (B). Trees of MLB- and VA-AstVs were drawn based on approximately 250 nt of 3’- (A-2, A-4, B-2, and B-4) and approximately 250 nt of 5’-end (A-3, A-5, B-3, and B-5) of amplicons. Phylogenetic clusters are collapsed for simplification. Bootstrap values greater than 650 among 1,000 replicates are shown on each branch. GenBank accession numbers of referential strains are as follows, CAstV type 1: L23513; -2: L13745; -3: AF141381; -4: DQ344027; -5: DQ028633; -6: GQ495608; -7: AF248738; - 8: AF260508; MLB-AstV type 1: FJ222451; - 2: JF742759; - 3: JX857870, VA-AstVs type 1: FJ973620; - 2: GQ415560; - 3: JX857868; - 4: JX857869; - 5: KJ65612, and FAstV: KM017742. GenBank accession numbers of outgroup strains are as follows, sheep AstV: Y15937; ovine AstV: NC002469; mink AstV: NC004579; porcine AstV: GQ91477; California sea lion AstV: JN42035; bat AstV: EU847155; turkey AstV: NC002470; and chicken AstV: JF832365.

|  |   **A1** |
| --- | --- |
|  | **** |
|  |   **A2** |
|  |   **A3** |
|  | ****  **A5**  **A4** |
|  |  |

|  | ****  **A6** |
| --- | --- |
|  |   **A7** |
|  |   **A8** |

|  |  |
| --- | --- |
|  |   **B2**  **B1** |

**Fig. S2.** Simplot (left) and bootscan (right) analysis of CAstV OTUs, including FAstV-like OTUs, identified from U.S. samples (A) and MLB-AstV OTUs identified from Nepal samples (B). OTUs supposed to be recombinants by the phylogenetic analysis (Fig S1. A-1 and B-3) were subjected to the analysis here. Representative OTUs resulted in Bootstrap values over 70% among 100 replicates in the bootscan analysis were considered significant and shown here. Names of OTUs used as a query sequence in each analysis are shown on upper side of the figures by the following manner, “Origin of the sample (-“type of wastewater”)” -“year or month of sample collection”-“OTU number”. Regarding wastewater samples, “A” and “B” indicate places of WWTP and “in” and “out” indicate influent and treated effluent, respectively. GenBank accession numbers of referential strains are as follows, CAstV type 1: L23513; -2: L13745; -3: AF141381; -4: DQ344027; -5: DQ028633; -6: GQ495608; -7: AF248738; - 8: AF260508; MLB-AstV type 1: FJ222451; - 2: JF742759; - 3: JX857870, and FAstV: KM017742. Window size and step size for each analysis were set as 100 and 20 nt, respectively. “Nucleotide position” shown on x-axis corresponds to CAstV type 1 strain Oxford and MLB-AstV type 1 (GenBank accession number: L23513 and FJ222451, respectively). Arrows and lines indicate locations of ORF1b and ORF2.

**SUPPLEMENTAL TABLE**

**Table S1. Numbers of sequencing reads obtained by the amplicon sequencing and those mapped to each human AstV reference sequences.**

|  |  |  |  | CAstV |  |  | MLB-AstV |  |  | VA-AstV |  |
| --- | --- | --- | --- | --- | --- | --- | --- | --- | --- | --- | --- |
| Sequencing Run | Number of sample | Total read counts |  | Number of positive sample | Number of reads mapped |  | Number of positive sample | Number of reads mapped |  | Number of positive sample | Number of reads mapped |
| 1 | 24 | 15,339,630 |  | 22 | 1,875,145 |  | 7 | 185,136 |  | 20 | 482,389 |
| 2 | 15 | 20,798,762 |  | 15 | 1,976,474 |  | 0 | 0 |  | 3 | 74,084 |
| 3 | 6 | 43,819,176 |  | 6 | 6,516,281 |  | 0 | 0 |  | 0 | 0 |
| Total | 54 | 79,957,568 |  | 43 | 10,367,900 |  | 7 | 185,136 |  | 23 | 556,473 |

**Table S2. Primers used in this study**

| **Target** | **Assay** | **Primer name** | **Function** | **Sequence (5' → 3')*^a^*** | **Location*^b^*** | **Reference** |
| --- | --- | --- | --- | --- | --- | --- |
| CAstV | First round PCR | AHAstVF1 | Sense primer | AATCACTCCATGGGAAGCTCCT | 4139-4160 | Hata et al., 2014 |
|  | First round PCR | AHAstVR1 | Antisense primer | CCTARCGCYTGCACDGG | 4697-4713 | Hata et al., 2014 |
|  | Second round PCR | AHAstVF2 | Sense primer | CAGAAGAGCAACTCCATCGCAT | 4280-4301 | Hata et al., 2014 |
|  | Second round PCR | AHAstVR2 | Antisense primer | GTRCTYCCWGTAGCRTCCTTAAC | 4664-4686 | Hata et al., 2014 |
| MLB-AstV | First and second round PCR | SF0073 | Sense primer | GAYTGGACWCGATTTGATGGTAC | 3110-3132 | Finkbeiner et al., 2009 |
|  | First round PCR | AHMLBR1 | Antisense primer | CAGGYTTAGGCCCAGTTGTA | 4016-4035 | Hata et al., 2015 |
|  | Second round PCR | AHMLBR2 | Antisense primer | CGAGTGAAGCGCCTTGGTAAG | 3778-3798 | Hata et al., 2015 |
| VA-AstV | First round PCR | AHVAF1 | Sense primer | TATGGGAARCTCCTTTGCTAYCGC | 4025-4048 | Hata et al., 2015 |
|  | First round PCR | AHVAR1 | Antisense primer | ARTTTCTTGACAAACCACCAWCC | 5211-5233 | Hata et al., 2015 |
|  | Second round PCR | AHVAF2 | Sense primer | ATGCTGGATAGRCTTTGGAGGG | 4172-4193 | Hata et al., 2015 |
|  | Second round PCR | AHVAR2 | Antisense primer | SCTCCCTCTTCATTKGTRTCTGT | 4812-4834 | Hata et al., 2015 |

*^a^*: Mixed base in degenerate primer is as follows, R stands for A or G; Y stands for C or T; D stands for A, G, or T; W stands for A or T; S stands for G or C; K stands for T or G.

*^b^*: Corresponding nucleotide position of HAstV-1 strain Oxford, MLB-AstV1, and VA-AstV1 (accession number: L23513, FJ222451, and FJ973620, respectively).
